# Navigational Efficiency of Nocturnal *Myrmecia* Ants Suffers at Low Light Levels

**DOI:** 10.1371/journal.pone.0058801

**Published:** 2013-03-06

**Authors:** Ajay Narendra, Samuel F. Reid, Chloé A. Raderschall

**Affiliations:** 1 ARC Centre of Excellence in Vision Science, Research School of Biology, The Australian National University, Canberra, Australian Capital Territory, Australia; 2 EMBL/CRG Systems Biology Unit, Centre for Genomic Regulation, Barcelona, Spain; Lund University, Sweden

## Abstract

Insects face the challenge of navigating to specific goals in both bright sun-lit and dim-lit environments. Both diurnal and nocturnal insects use quite similar navigation strategies. This is despite the signal-to-noise ratio of the navigational cues being poor at low light conditions. To better understand the evolution of nocturnal life, we investigated the navigational efficiency of a nocturnal ant, *Myrmecia pyriformis*, at different light levels. Workers of *M. pyriformis* leave the nest individually in a narrow light-window in the evening twilight to forage on nest-specific *Eucalyptus* trees. The majority of foragers return to the nest in the morning twilight, while few attempt to return to the nest throughout the night. We found that as light levels dropped, ants paused for longer, walked more slowly, the success in finding the nest reduced and their paths became less straight. We found that in both bright and dark conditions ants relied predominantly on visual landmark information for navigation and that landmark guidance became less reliable at low light conditions. It is perhaps due to the poor navigational efficiency at low light levels that the majority of foragers restrict navigational tasks to the twilight periods, where sufficient navigational information is still available.

## Introduction

Insects are active at different times of the day. At night, the light intensity is nearly 6–11 orders of magnitude dimmer than in the day [1]. To cope with this dramatic change in light intensity insects require distinct visual adaptations. To increase their optical sensitivity, most nocturnal insects have superposition eyes (e.g., moths), where light from several lenses is superimposed on to a single photosensitive structure, the rhabdom [1], [2], [3], [4]. However, nocturnal hymenopteran insects (e.g., ants, bees, wasps) have apposition eyes, where light reaches the rhabdom through a single lens, thus being less sensitive compared to the superposition eyes. To overcome this reduced sensitivity, nocturnal hymenopterans increase their optical sensitivity by having larger lenses and wider photoreceptors compared to their diurnal relatives [1], [5], [6], [7], [8], [9], [10], [11]. Insects that are active in a wide range of ambient light levels cope with the variation in light intensities to some extent by increasing or decreasing the sensitivity of their visual system through pupillary mechanism [12].

Irrespective of the time at which insects are active, a common challenge faced by all animals is navigation. Both diurnal and nocturnal insects appear to use very similar navigation strategies [1]. To determine the compass direction, insects rely on the pattern of polarised skylight (diurnal: e.g., *Cataglyphis fortis* [13], *Apis mellifera* [14]; nocturnal: e.g., *Myrmecia pyriformis* [15], *Scarabaeus zambesianus* [16]), or on the visual landmark panorama (diurnal: *Apis mellifera*, *Cataglyphis fortis* [17], *Melophorus bagoti* [18], [19]; nocturnal: *Myrmecia pyriformis* [15], *Megalopta genalis* [20]). There is now growing evidence that diurnal insects also orient using the geomagnetic field [21]. While specific evidence for the use of geomagnetic field by nocturnal insects is not available, it is most likely used as an orientation cue by nocturnal migrating insects [22], [23]. To estimate the distance travelled flying insects integrate optic flow information (diurnal: *Apis mellifera* [24]; nocturnal: *Megalopta genalis* [25]) and walking insects such as ants use some form of a stride integrator [26]. Though the importance of colour in the context of navigation remains to be fully understood, it is clear that nocturnal insects similar to their diurnal counterparts use colour information for localisation even at the low starlight intensities that they operate at [27], [28], [29].

As light levels drop the available visual information for navigation becomes weaker, resulting in a poor signal-to-noise ratio. But the navigational requisites of diurnal and nocturnal animals remain similar. To explain the evolution of nocturnal life it is hence interesting to ask whether the navigational efficiency of nocturnal animals suffers at low light. The nocturnal Namibian spider, *Leucorchestris arenicola*, while navigating to its burrow, pause and stay still for upto 1s at the lowest light intensities at which they operate [30]. These pauses have been suggested to be a behavioural adaptation for low light to enable animals to collect enough light to detect coarse landscape structures. In the nocturnal sweat bee, *Megalopta* species, individual bees took longer to locate the nest in dim light compared to slightly brighter conditions [31]. The longer duration was due to their tortuous flight trajectories in contrast to the directed flights of individuals in slightly brighter conditions. The flight speed of the nocturnal sweat bee, *Megalopta genalis* was also found to be nearly five times slower than that of the diurnal bee, *Bombus terrestris* [25], indicating that their flight performance suffers at dim light. Walking in a straight line requires the use of an external compass [32]. Interestingly, both diurnal and nocturnal beetles seem to achieve this with similar accuracy in their ability to maintain a straight path by using a sun compass and moon compass respectively [33].

**Figure 1:**
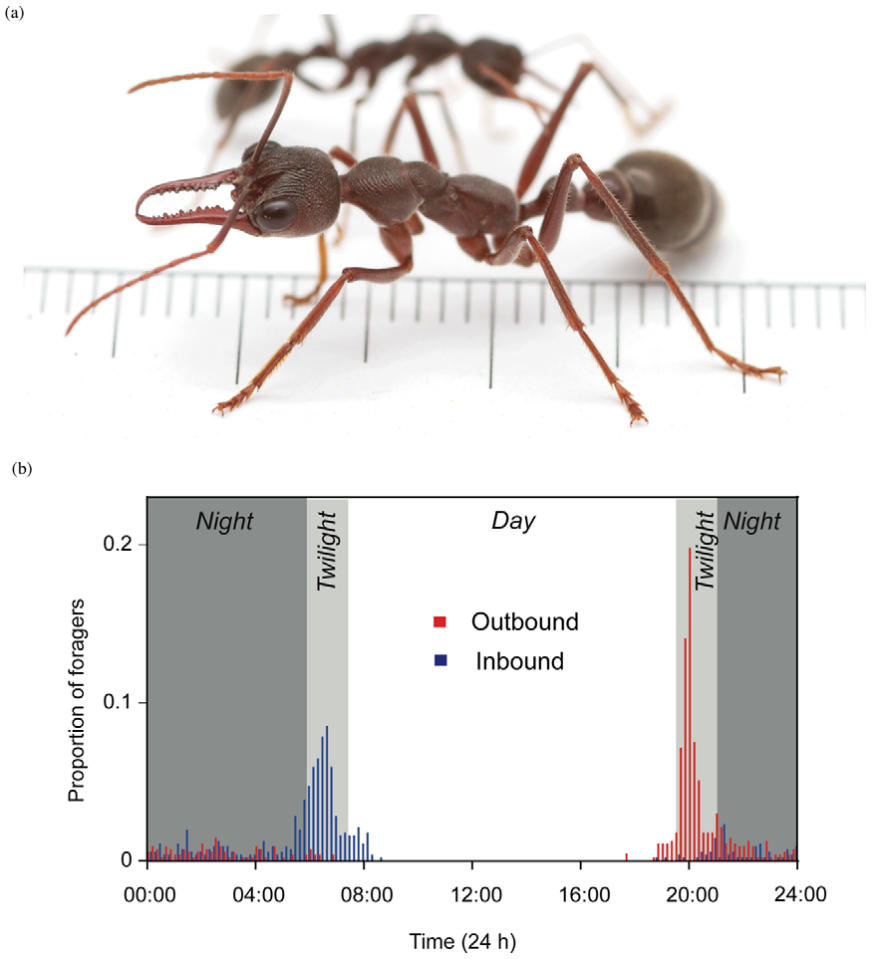
The study species, *Myrmecia pyriformis* and its daily activity rhythm. (a) The nocturnal bull ant, *Myrmecia pyriformis*. Graduations are in mm. (b) Activity rhythm of *M. pyriformis* on one summer day. Bars indicate the proportion of outbound (red) and inbound (blue) workers in 10-minute bins. Modified from Narendra [34].

We here study the nocturnal Australian Bullant, *Myrmecia pyriformis* Smith (Figure 1) to find out whether their navigational efficiency suffers at low light levels. This study species is appropriate for the question of interest for the following reasons. Firstly, in ants it is possible to record the entire path of individuals with sufficient accuracy under ecologically relevant conditions. Secondly, *M. pyriformis* are nocturnal foragers, where majority of the workers leave the nest in a narrow time-window of 40–60 minutes during the evening twilight (Figure 1b) [34]. Ants typically travel to nest-specific *Eucalyptus* trees on which they forage, with each individual carrying out only one foraging trip per day [35]. A small proportion of workers (10–12% of daily forager force) capture prey and attempt to return to the nest immediately in the dark. The majority of workers, however, return to the nest in the morning twilight after feeding on honeydew produced by sap-sucking insects. The ants thus present a natural scenario where animals tackle the task of navigation in a gradient of light intensities from dark, dim and bright conditions enabling us to investigate the navigational efficiency in these different light conditions. We first report that nocturnal Bullants pause for longer and walk slower as light levels drop. Next, we investigated homing abilities of ants in dark, dim and bright light conditions, where we measured homing success, path sinuosity and travel time. The nocturnal *M. pyriformis* workers rely heavily on visual landmark information for navigation [15] and hence we asked whether homing efficiency is affected by the difficulty in using landmark information.

## Materials and Methods

### Study location

This study was carried out at The Australian National University Campus Field Station, Canberra, Australia (35°16′50.14”S, 149°06′42.13”E) between 2009–2012, where we located five nests of *Myrmecia pyriformis* Smith. Forager forces varied between nests and between seasons. Thus to ensure we had sufficient numbers for each experiment we studied 3 nests (A, B, & C) all located within 30 m from each other. Ants were collected, marked for individual identification using model paint (Citadel, Games Workshop, UK) and released back to the nest 3–4 days before the start of experiments. Astronomical data were obtained from Geoscience Australia (http://www.ga.gov.au).

### Experimental procedure

#### Natural paths of ants (Nest C)

Ants from each nest typically head in a specific direction to a specific tree [15]. We tracked outbound paths of 9 individual ants from a single nest in three 30-minute bins after sunset. We noted the spatial location of pauses (>1 sec) along each individual ant's path and the time taken by individual ants to reach the tree.

#### Pausing behaviour and walking speed (Nest A, B)

We first identified the typical route ants took to travel from the nest to the tree. Along this path, at a distance of 1–2 m from the nest, we set up a video camera (SONY Handycam DCR-HC21E PAL) with a night-shot mode and infrared light sources and mounted it on a tripod. The camera zoom was set to film an area of 0.6×0.45 m to get the best resolution of individual ants. We recorded the paths of ants heading towards their foraging tree. A frame-by-frame video analysis was carried out to determine the head position of ants at every 400ms to measure the pause duration and walking speed (only time in motion was used) of individual ants. We measured ambient light levels using an ILT1700 radiometer with a SHD033 detector (Warsash Scientific). Light data were averaged over 20 seconds.

#### Homing accuracy at different light conditions (Nest B)

We followed foraging ants that left the nest in the evening twilight and arrived at the base of nest-specific *Eucalyptus* tree. Ants were individually captured at the base of the tree in foam-stoppered transparent Perspex tubes. They were fed with 10% sugar solution and prey and kept in ambient light conditions. Ants were individually released at the base of the same tree the next day in three time slots: dark (60–120 minutes before sunrise); dim (30 minutes on either side of sunrise); and bright condition (60–120 minutes after sunrise). These release times were chosen since it best matches the typical return times of the majority of the ants. We tracked the individual paths using a Differential Global Positioning System (for details see below), noted the time of release and the nest-entry for each ant.

#### Visually mediated homing in bright and dark conditions (Nest A)

We determined whether landmark guidance for *M. pyriformis* workers becomes less reliable at low light conditions. Although a majority of *M. pyriformis* workers head out foraging in the evening twilight, a few leave the nest before sunset [34]. We made use of this variability to capture ants at the base of the foraging tree before and after sunset. Captured ants were fed with sugar and prey, transferred in the dark to a location 12 m perpendicular to the normal foraging direction and released within 10 minutes of being captured. Ants were tracked until they reached the nest or for 50 minutes. When possible we kept note of the final destination of these ants, typically the nest or the tree.

#### Tracking technique

Ant paths were tracked by placing small markers at every 5 cm behind a walking ant, carefully avoiding disturbing the ants' progress. The marked path was later recorded using a Differential Global Positioning System (DGPS, NovAtel Inc., Canada). The DGPS set-up consisted of a base station antenna (GPS-702-GG L1/L2, GPS plus GLONASS), a base station receiver (FLEXPAK-V2-L1L2-G GPS plus GLONASS RT-2), a rover antenna (ANT-A72GLA-TW-N (532-C) and a rover receiver (OEMV-2-RT2-G GPS plus GLONASS). The stationary reference or base station calculates corrections for a mobile rover antenna, the position of which is determined with centimetre accuracy on a local scale, in this case a 30 m radius. We mounted the stationary base station electronics and antenna on a tripod to integrate position readings over 30 minutes. The rover receiver electronics were carried on a backpack and connected to the rover antenna that was mounted at the end of a long, hand-held stick. This was moved along the flag-marked path. The base station and rover communicate through a radio link, where corrections are exchanged. We monitored the errors constantly and tracked paths only when errors were less than 10 cm. Northing, Easting and Height coordinates in metres, together with the standard deviations of position error estimates were recorded at 1s intervals with a laptop and extracted with a custom-written Matlab program (© Jan M. Hemmi).

We tested whether pause duration and walking speed of ants was affected by ambient light levels and also determined whether there was a nest effect using a linear regression model in R computing environment [36]. The effect of light condition on the different measures of navigational accuracy was determined by a Kruskal-Wallis analysis. Where required a Dunn's multiple comparison test was carried out. Path straightness was determined by E_max_ [32], with higher E_max_ values indicating straighter paths. E_max_ is a dimensionless value that indicates the maximum possible expected displacement, which is expressed as a function of the number of steps. Circular statistics and plotting was carried out in R computing environment [36].

## Results

### Natural paths of ants

**Figure 2:**
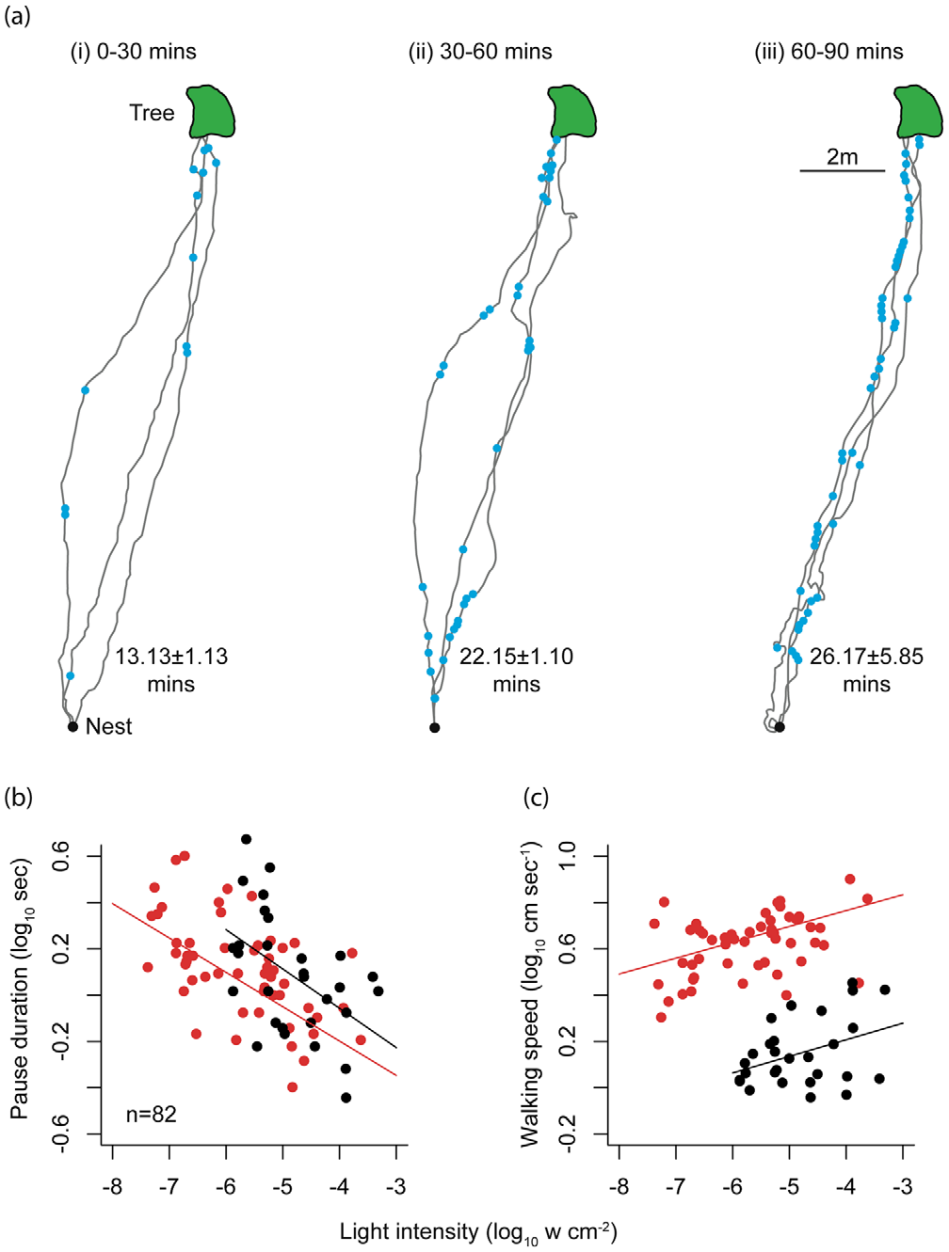
Effect of ambient light intensity on walking speed and pause duration and frequency in the nocturnal ant, *Myrmecia pyriformis*. (a) Example trajectories of 9 ants and pauses they made (blue dots) on their foraging route at (i) 0–30 minutes after sunset, (ii) 30–60 minutes after sunset and (iii) 60–90 minutes after sunset. Time taken to travel from the nest to tree is shown as means±SD. (b) Pause duration and (c) walking speed of animals plotted against light levels. Two nests were studied and are indicated as red and black. Regression lines for each dataset are shown.

Initial observations showed that ants that left the nest early (0–30 minutes after sunset) reached the tree faster (13.13±1.13 mins; n = 3) than those that left 60–90 minutes after sunset (26.17±5.85 mins; n = 3; Figure 2a). Not only did the late ants appear to walk more slowly, they also stopped more frequently compared to the early foragers (Figure 2a; blue circles along each path). This indicated that navigational efficiency of *M. pyriformis* may suffer at low light. Since pause duration and walking speed was difficult to accurately measure from these observations, we addressed these questions in a separate experiment reported below.

### Pausing behaviour and walking speed

We recorded pauses ranging from 0.4–4.0 seconds (n = 53, Nest A) and 0.36–4.72 seconds (n = 29, Nest B). Pause duration increased significantly with decrease in light intensity (P<0.001, t = −4.328) and there was no significant difference between the two nests (r^2^ = 0.28, P = 0.63, t = −0.482, Figure 2b). Ants walked more slowly as it became darker and the walking speeds (i.e., time in motion) decreased from 7.97 cm s^−1^ to 2.01 cm s^−1^ at nest A (red dots) and from 2.84 cm s^−1^ to 0.90 cm s^−1^ at nest B (black dots). Walking speed of ants decreased with decrease in light intensity (r^2^ = 0.78, P<0.001, t = 3.140, Figure 2c). Ants from nest B (black circles in Figure 2c) walked more slowly than those from nest A (P = 0.017, t = −2.437). This difference is most likely due to the micro environmental factors such as surface texture and undergrowth [15].

### Homing accuracy at different light conditions

**Figure 3:**
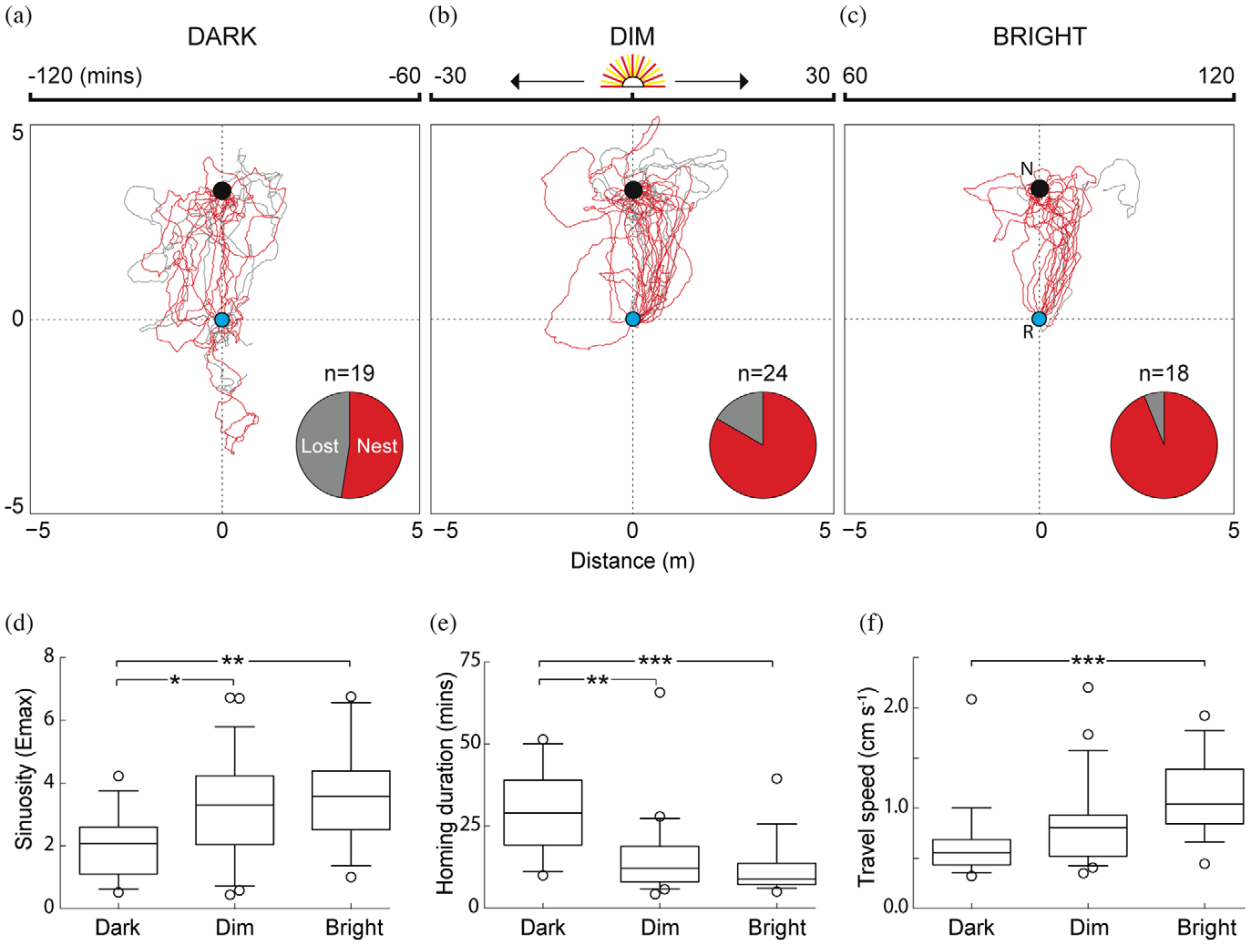
Homing success and navigational efficiency of the nocturnal bull ant, *Myrmecia pyriformis*. Top row: Homing paths of ants released at the base of their foraging tree (R, blue circle) to the nest (N, black circle) in three one-hour slots: (a) Dark: 60–120 minutes before sunrise, (b) Dim: 30 minutes on either side of sunrise, (c) Bright: 60–120 minutes after sunrise. Red: ants that successfully returned to the nest; grey: ants that did not return to the nest within 50 minutes of tracking. Bottom row: (d) Sinuosity of all paths: the larger the E_max_ value the straighter are the paths; (e) homing duration of successful ants (difference between the times of release and nest entry); (f) travel speed of all ants including pauses. Sector graphs show the proportion of ants that reached the nest within the recording duration of 50 minutes. Significance codes: *p<0.05; **p<0.01; ***p<0.001.

When ants were released at their point of capture at different light conditions their homing success decreased, their paths became less straight and ants took 2–3 times longer to return home as light levels dropped. The proportion of animals that returned home in the 50 minute recording duration was lowest in the dark (52.6%, n = 19), increased in the dim (83.33%, n = 24) and was maximum in the bright condition (93.75%, n = 18; Figure 3a, 3b, 3c; red paths  =  successful; grey paths  =  unsuccessful). The sinuosity of the paths was significantly different between the dark, dim and bright conditions (P<<0.01, KW = 10.21; Figure 3d). Paths were least straight in the dark and became increasingly straighter in the dim (P<0.05, Dunn's test) and bright conditions (P<0.01). Homing duration of successful ants also differed significantly between the three conditions (P<0.001, KW = 14.89; Figure 3e). Ants took the longest time to reach the nest in the dark (29.22±3.7 mins), compared to the dim (15.52±2.9 mins) and bright (11.79±2.1 mins) conditions. A comparison of travel speed in dark, dim and bright conditions revealed the effect of low light (P<0.001, KW = 18.69; Figure 3f) with ants being slowest in the dark (0.62±0.09 cm s^−1^; means±SE), and fastest in the bright (1.12±0.09 cm s^−1^) conditions. The ‘lost’ ants released in the dark eventually returned to the nest once it got bright, but well beyond the 50 minutes of recording duration per individual.

### Visually mediated homing in bright and dark conditions

**Figure 4:**
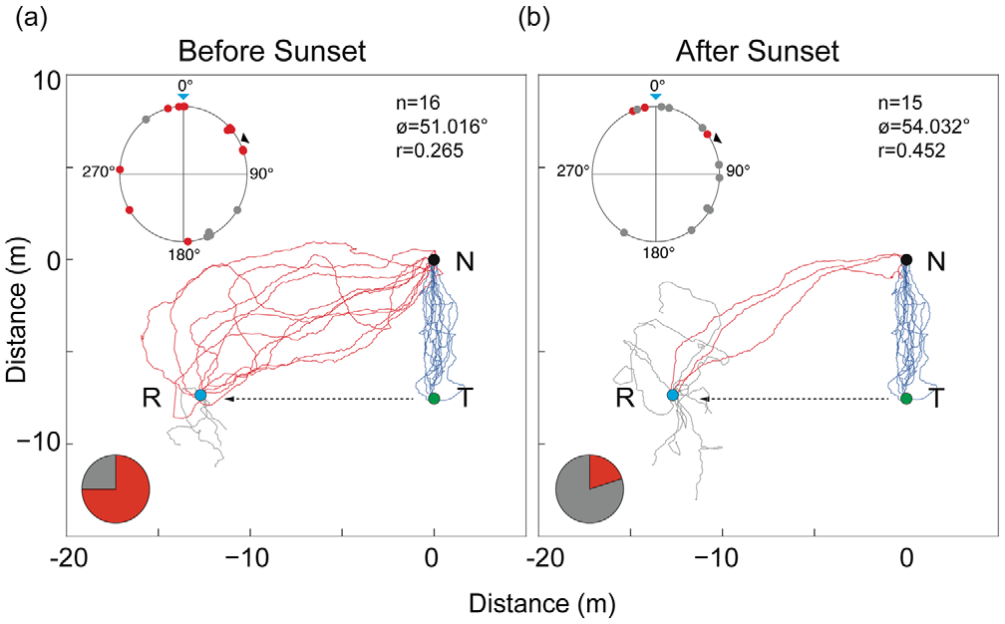
Visually mediated homing in bright and dark conditions. Responses to displacement (a) before sunset, and (b) after sunset. Blue: outbound paths of 15 individual ants from nest (N) to tree (T). Ants were captured at the tree (T), fed and transferred in the dark to R (12 m in a direction perpendicular to the normal foraging direction). Red: ants that successfully returned to the nest; grey: ants that did not return to the nest. Circular plots indicate initial bearing of ants at 0.5 m from release. Black arrow: true nest direction; blue arrow: fictive nest direction based on a path integrator (blue arrow) is shown. Sector graphs show the proportion of ants that reached the nest within the recording duration of 50 minutes.

Ants individually travelled in a narrow corridor from the nest to their main foraging tree (blue paths; Figure 4a). When displaced lateral to their typical foraging route, the proportion of ants that found the nest, within the recording duration, was higher before sunset (75%, red paths, Figure 4a) than after sunset (20%, red paths, Figure 4b). The initial mean heading direction of ants before sunset (ø = 51.016°) and after sunset (ø = 54.033°) was close to the true nest direction (ø = 60°). But since the orientation of ants was distributed uniformly around a circle (Rayleigh's test of uniformity; before sunset: Z = 1.198, p = 0.30; after sunset: Z = 2.655, p = 0.07) there seemed to be no specific directional preference, which is further emphasised by the very short length of the mean vectors (‘r’ in Figure 4a, 4 b). Among the successful ants, the initial orientation of ants was directed either towards the true nest (60°), or towards the fictive nest based on a celestial compass (0°) and in some cases even opposite to the true nest direction. However, most successful ants corrected their heading within 3–4 m from the release and headed directly to the nest. Not a single ant relied on the path integrator to travel the entire home vector, either before or after sunset.

## Discussion

The majority of the workers of *M. pyriformis* travel to their favourite tree and back to the nest in the evening and morning twilight respectively [34]. A small proportion of ants carry out this task of navigation at night. We hence asked whether the navigational efficiency of animals changes at different light conditions. We found that as light levels dropped, ants paused for longer durations and walked more slowly to reach their goal. Displacement experiments showed that in both bright and dark conditions, ants relied mainly on visual landmark information for homing and not on path integration and that landmark guidance became less reliable in low-light conditions.

Many insects have adopted a crepuscular to nocturnal lifestyle, yet for navigation they still have to rely on visual information. This is particularly true for the solitary foraging ants (e.g., *Cataglyphis*, *Melophorus*, *Myrmecia*, *Harpegnathos*). To partially account for the low light levels at which they are active, workers of *M. pyriformis* have evolved visual adaptations to increase their photon capture [5], [10]. Their lens diameters are nearly 3 times larger and photoreceptors nearly 3 times wider compared to day-active species [5], [10], [37]. These adaptations increase the optical sensitivity of the night-active ants by at least 27 times [10], quite similar to the increase in optical sensitivity in the nocturnal halticid bee, *M. genalis* [20], [38]. However, these optical adaptations alone are not sufficient to support visual navigation and it has been suggested that insects need to engage in two forms of neural summation to improve vision in low light [8], [39], [40], [41]: spatial summation (pooling signals from neighbouring photoreceptors) and temporal summation (increase in integration time). The increasing duration of pauses we observed in *M. pyriformis* as light levels dropped (Figure 2a, 2 b) is a possible behavioural strategy whereby animals could increase the integration time to capture more light and to generate a brighter view of the world. A similar function has been attributed to the pausing behaviour of the Namibian spider, *L. arenicola*. These spiders paused for 1s at the lowest light intensities they operate at, and individuals travelled <2 meters between successive pauses [30]. The short pause durations in the nocturnal spider may very well be due to their highly sensitive optics compared to the apposition compound eyes of ants, which perhaps requires longer pause durations to get a similar brighter view of the world.

The navigational efficiency of the nocturnal workers of *M. pyriformis* suffers at low light conditions. As light levels dropped, the walking speed of animals decreased, their ability to walk in a straight line was affected, time taken to reach their goal increased and the proportion of animals that successfully returned to the nest decreased not only along their normal foraging corridor (Figure 3) but also following a local displacement (Figure 4). At low light conditions animals could not compensate for a local displacement as well as animals in bright light conditions (Figure 4). Animals appeared to rely predominantly on visual landmark information rather than path integration in both bright and dim conditions, but landmark guidance appears to be not sufficient to compensate for the displacement in dim light conditions. Given these findings, it is interesting to note that most workers of *M. pyriformis* time their foraging excursions to a narrow time window in the evening twilight, which most likely offers sufficient navigational information.

It is also relevant to note that in ants and other insects, walking speed is typically affected by temperature [42], but this is unlikely to explain the differences in navigational efficiency that workers of *M. pyriformis* exhibited. We have previously shown that workers of *M. pyriformis* retain their nocturnal habits throughout the year and thus encounter a wide range of temperatures ranging between 5–30°C [34]. From laboratory studies we know that within this temperature range the walking speed of workers remains fairly constant and increases only beyond 35°C [43]. Temperatures encountered by ants during our study were between 8–17°C, which was well within the range where walking speed of ants remain less affected by temperature. Hence it is unlikely that temperature variation affected the walking speed of ants in our study. On a daily basis, workers of *M. pyriformis* attempt to return home upon capturing prey throughout the night. Hence motivation for finding the nest at different light conditions is very unlikely to be a reason for the navigational differences we found at the different light conditions.

Workers of *M. pyriformis* adhere to a crepuscular/nocturnal foraging period throughout the year, with their activity primed by light levels around sunset and most likely sunrise time [34]. Despite being able to find home faster at brighter light levels, they do not navigate or forage in the day. This raises the question of why these ants remain so stubbornly night-active throughout the year.
